# ‘Proactive’ use of cue-context congruence for building reinforcement learning’s reward function

**DOI:** 10.1186/s12868-016-0302-7

**Published:** 2016-10-28

**Authors:** Judit Zsuga, Klara Biro, Gabor Tajti, Magdolna Emma Szilasi, Csaba Papp, Bela Juhasz, Rudolf Gesztelyi

**Affiliations:** 1 Department of Health Systems Management and Quality Management for Health Care, Faculty of Public Health, University of Debrecen, Debrecen, Nagyerdei krt. 98, 4032 Hungary; 2 Department of Pharmacology, Faculty of Pharmacy, University of Debrecen, Debrecen, Nagyerdei krt. 98, 4032 Hungary; 3 Department of Pharmacology and Pharmacotherapy, Faculty of Medicine, University of Debrecen, Debrecen, Nagyerdei krt. 98, 4032 Hungary

**Keywords:** Model-based reinforcement learning, Proactive brain, Bellman equation, Reward function, Policy function, Cue-context congruence

## Abstract

**Background:**

Reinforcement learning is a fundamental form of learning that may be formalized using the Bellman equation. Accordingly an agent determines the state value as the sum of immediate reward and of the discounted value of future states. Thus the value of state is determined by agent related attributes (action set, policy, discount factor) and the agent’s knowledge of the environment embodied by the reward function and hidden environmental factors given by the transition probability. The central objective of reinforcement learning is to solve these two functions outside the agent’s control either using, or not using a model.

**Results:**

In the present paper, using the proactive model of reinforcement learning we offer insight on how the brain creates simplified representations of the environment, and how these representations are organized to support the identification of relevant stimuli and action. Furthermore, we identify neurobiological correlates of our model by suggesting that the reward and policy functions, attributes of the Bellman equitation, are built by the orbitofrontal cortex (OFC) and the anterior cingulate cortex (ACC), respectively.

**Conclusions:**

Based on this we propose that the OFC assesses cue-context congruence to activate the most context frame. Furthermore given the bidirectional neuroanatomical link between the OFC and model-free structures, we suggest that model-based input is incorporated into the reward prediction error (RPE) signal, and conversely RPE signal may be used to update the reward-related information of context frames and the policy underlying action selection in the OFC and ACC, respectively. Furthermore clinical implications for cognitive behavioral interventions are discussed.

## Background

Reinforcement learning is a fundamental form of learning where learning is governed by the rewarding value of a stimulus or action [1, 2]. Concepts of machine learning formally describe reinforcement learning of an agent using the Bellman equation [3], where the value of a given state (reached following a specific action) is:

$$V^{\pi } \left( s \right) = \mathop \sum \limits_{a \in A\left( s \right)} \pi \left( {s,a} \right)\mathop \sum \limits_{{s^{\prime}}} T\left( {s,a,s^{{\prime }} } \right) \cdot \left[ {R\left( {s,a,s^{{\prime }} } \right) + \gamma V^{\pi } \left( {s^{{\prime }} } \right)} \right]$$

with V^π^(s): value of state “s”; aϵA(s): action set available to the agent in state “s”; π(s, a): policy denoting the set of rules governing action selection; T(s, a, s′): state transition (from s to s′) probability matrix; R(s, a, s′): reward function; γ: discount factor; V^π^(s′): value of state following state “s” (i.e. value of state “s′”).

The Bellman equation is a central theorem in reinforcement learning, it defines the value of a given state as the sum of the immediate reward received upon entering a state and the discounted value of future states that may be obtained starting from the current state. The value of state is determined by agent related attributes (action set, policy and γ discount factor), the agent’s knowledge of the environment (described by the reward function) and environmental factors hidden to agent (given by the transition probability). Accordingly, while the set of actions and policy are inherent to the agent, the reward function and the transition probabilities are characteristics of the environment, by definition they are beyond the agent’s control. Thus, the need to obtain information about these two functions stands in the focus of reinforcement learning problems (for a more elaborate overview, see: [4]). This may be done by either building a world model that compiles the reward function and the transition probabilities or omitting the use of a model. In the latter case, the agent obtains information about its environment by trial and error and computes estimates of the value of states or state-action pairs, in a way that estimates are cached [3, 5]. These two distinct approaches to solve reinforcement learning problems are embodied by the concepts of model-based and model-free reinforcement learning, respectively. This distinction carries several implications about learning and updating the value of state as well as concerning the ability to carry out predictions, forward-looking simulations and optimization of behavior. Model-free learning, by omitting the use of a model, provides an estimate of the value function and/or the policy by use of cached state or state-action values that are updated upon subsequent learning. Conversely, predictions also concern the estimated values [4]. Model-based learning, however is characterized by use of a world model [6], therefore direct experience is used to obtain the reward function and the transition probabilities of the Bellman equation. Herein, learning is used to update the model (as opposed to model-free learning, where learning serves to update the cached estimated value of state). Generally, model-based reinforcement learning problems use the model to conduct forward-looking simulations for the sake of making predictions and/or optimizing policy in a way that the cumulated sum of the reward is maximized in the long term. Nevertheless, under the assumption that the Bellman equation is appropriate to describe model-based reinforcement learning, the recursive definition of the state value (e.g. a value of a state incorporates the discounted value of the successive state, as well as the successive state to that, so forth) should be acknowledged. This implies that under model-based reinforcement learning scenarios, predictions (e.g. attempts to determine the value of state) are deduced from information contained in the model. Thus a relevant issue for model-based reinforcement learning, concerns the world model underlying predictions, is updated. Former reports have implicated cognitive efforts [7] or supervised learning as possible mechanisms for updates, nonetheless further insight is needed.

The neurobiological substrate of model-free reinforcement learning is well rooted in the reward prediction error hypothesis of dopamine, i.e., upon encountering unexpected rewards or cues for unexpected rewards, ventral tegmental area (VTA) dopaminergic neurons burst fire. This leads to phasic dopamine release into the synaptic cleft that, by altering synaptic plasticity, may serve as a teaching signal underlying model-free reward learning [8, 9]. This phasic dopamine release is considered to be the manifestation of a reward prediction error signal computed as the difference between the expected and actual value of the reward received, and it drives model-free reinforcement learning [2, 8]. While the model-free learning accounts are well characterized, the notions relating to how the brain handles model-based reinforcement learning are vague. In addition to the question of updating the world model posited before, resolution of other critical unresolved issues await including: how an agent determines the relevant states and actions given the noisy sensory environment, how are the relevant features of states determined by the agent, how can an agent effectively construct a simplified representation of the environment in a way that the complexity of state-space encoding is reduced [10]?

In the current paper, building on the theory of the ‘proactive brain’ [11, 12] and a related proactive framework that integrates model-free and model-based reinforcement learning [4], we expand the neurobiological foundations of model-based reinforcement learning. Previously, using the distinction for model-based and model-free learning and taking the structural and functional connectivity of neurobiological structures into consideration, we offered an overview of model-free and model-based structures [4]. According to our proactive account, the ventral striatum serves as a hub that anatomically connects model-free (pedunculo-pontine-tegmental nucleus (PPTgN) and VTA) and model-based [amygdala, hippocampus and orbitofrontal cortex (OFC)] structures, and integrates model-free and model-based inputs about rewards in a way that value is computed (the distinction between reward and value must be noted at this point [4]). Additionally, based on the neuroanatomical connections between model-based and model-free structures and experimental findings of others, we have also suggested that these systems are complementary in function and most likely interact with each other [4, 10, 13–15]. Based on the structural connectivity of the ventral striatum and other, model-based structures (hippocampus, medial OFC (mOFC), amygdala) [16], as well as their overlap with the default mode network [17, 18], we further suggested that the model used for model-based reinforcement learning is built by the default mode network [4].

In the present concept paper, the proactive brain concept is further described to show how the brain creates simplified representations of the environment that can be used for model-based reinforcement learning, and how these representations are organized to support the identification of relevant stimuli and action. Moreover we further expand our integrative proactive framework of reinforcement learning by linking model-based structures [the OFC, the anterior cingulate cortex (ACC)] to the reward and the policy function of the Bellman equation, respectively, providing a novel mathematical formalism that may be utilized to gain further insight to model-based reinforcement learning. Accordingly based on our proactive framework and works of others, we propose that OFC computes the reward function attribute of the Bellman equation, a function, that integrates state-reward contingencies and state-action-state’ transactions (e.g. how executing an action determines transitioning from one state to the other one). Furthermore, using the proactive brain concept we suggest that the mOFC formulates reward expectations based on cue-context congruence by integrating cue (amygdala) and context (hippocampus) related input while the lateral OFC (lOFC) contributes to action selection by solving the credit assignment problem. Moreover we propose that ACC a key structure for action selection, computes the policy function of the Bellman equation by capturing reward history associated with previous action. Additionally, using fundamental concepts of the proactive framework, we offer testable hypotheses based on the interaction between model-based and model-free systems. On one hand, we propose that the function of VTA dopaminergic neurons may be altered by manipulating OFC glutaminergic input. On the other, we propose that the model used by model-based reinforcement learning is updated by the interaction of the model-free and model-based accounts as model-free dopaminergic prediction error signals are able to influence the function of several model-based structures (OFC, hippocampus, amygdala, ACC, insular cortex).

## Results

### The proactive brain builds a model of the environment

A key issue of model-based learning concerns to how the brain creates the internal representations of the environment, thus how it segments and identifies relevant stimuli, contexts and actions [10]. The world model must represent the salient features of the external and internal (interoceptive, viscerosensory, affective and cognitive) environment. Previously, building on the proactive brain concept coined by Bar [19], we have proposed that model-based learning utilizes association-based context frames to build its world model, upon which forward looking mental simulations and predictions may be formulated [4]. A key to this concept is the creation of context frames. This is done by arranging stimuli (e.g. unconditioned stimuli and their conditioned cues) and their contexts into context frames. Contexts encompass internal [cognitive/affective (including reward-related), interoceptive (physiological and neurohumoral)] and external (spatial, temporal, social or cultural) settings [20, 21], thus context frames contain a priori information about the scalar value of reward [22]. (Context frames have been also referred to as schemata or scripts [19, 23]).

Context frames contain contextually associated information as an average of similar contexts containing typical, generic representations and constant features. Thus they include the probable stimuli and cues clustered together, their relationships and their affective and reward value [19, 23]. Furthermore, context frames come to signal cue-context associations reflecting statistical regularities and a lifetime of extracting patterns from the environment (related to contingencies, spatial locations, temporal integration, etc.) [23, 24]. Organization of context frames enables rudimentary cue- or context-related information to retrieve the most relevant context frame from memory, by means of associative processes [23, 24]. Furthermore it helps to cope with ambiguity and uncertainty, as coarse contextual information is sufficient to activate the most relevant context frame, which may assist in predicting the most probable identity of the cue. This stands to the extent that contextual retrieval may be used to disambiguate the cue-reward relationship (in context discrimination tasks [25]).

We feel that use of context frames for modelling the environment offers a sound hypothesis regarding how the agent generates a simplified representation of the environment, and how it defines the relevant states used for model-based learning. Furthermore it provides a feasible mechanism to identify relevant states and actions regardless the noise encountered in the sensory environment. (It should be noted that these context frames are conceptually similar to (if not equivalent with) the states of the reinforcement learning framework [3, 26], and they also correspond with the ‘task space’ described by others [27]).

The environment is transformed into context frames by means of cue and context conditioning. Cue and context conditioning are two concepts familiar to Pavlovian learning, with cue conditioning being the central paradigm [28]. Nonetheless, significance of context conditioning (emerging as context’s rising role in shaping cognitive and affective processes) is being increasingly acknowledged [20]. Cue and context conditioning are done by parallel but richly interconnected systems, with prior research pinpointing the amygdala as a neural substrate that is the prerequisite for affective processing of a stimuli as well as for cue-conditioning (e.g. forming associations between cues and primary reinforcers) [29, 30]. Furthermore, amygdaloid input, representing subcortical inferences pertaining to the affective and motivational value of the stimulus, is incorporated into decisions by function of the OFC [31]. Hippocampus assumes a central role in context conditioning, as the hippocampal area is critical for providing complex representation of signals; and its link with the OFC has been implicated in the integration of declarative representations with other information to guide behavior [20, 29]. Additionally, recent observations showed an interaction between the hippocampus and OFC in support of context-guided memory [32]. Furthermore using this proactive framework, we have previously proposed that the basolateral amygdala computes cue-reward, while the hippocampus forms context-reward contingencies, respectively [4]. Summarizing, using the proactive framework for reinforcement learning, we lay out a representational architecture based on cue-context associations and propose that OFC has a central role in computing state-reward contingencies based on the cue-reward, and context-reward information that are delivered by the amygdala and hippocampus, respectively.

### The orbitofrontal cortex compounds the reward function attribute of the Bellman equation

The central proposition of the current article is that the reward function of the Bellman equitation ‘R(s,a,s′)’, descriptive of the agent’s knowledge of the environment, is built by the OFC with distinct parts assuming well differentiated roles (the medial and lateral part contributing to state-reward contingency and state-action-state contingencies, respectively). The reward function contains information about the scalar value of reward and the state-action-state’ contingencies (e.g. it informs about a successive state following action ‘a’). Using the proactive model of reinforcement learning and experimental findings of others, we propose that the mOFC integrates cue- and context-based pieces of information provided by the amygdala and hippocampus, respectively, to assess cue-context congruence. Based on cue-context congruence, it identifies the context frame most relevant for a given state, to extract information regarding reward expectations. Furthermore, we provide insight that the lOFC may contribute to the credit assignment domain of action selection by having access to information about state-action-state’ contingencies. To support our proposal, relevant theoretical and experimental findings of others will be presented in the following sections.

The integrative function of OFC is well in agreement with its anatomical position, as it complies input from all sensory (e.g. visual, auditory etc.) modalities and subcortical (e.g. hippocampus, amygdala, ventral striatum, VTA, etc.) areas [33]. In line with this central position is OFC’s ability to integrate concrete and abstract multisensory perceptual input with memories about previous stimuli, state transactions as well as affective and incentive value of associated outcomes [27, 29, 32].

Hypotheses indicating that the OFC represents models for reinforcement learning has been formulated by others as well. Similar to our proposition is the concept of Schoenbaum and colleagues, who laid out a sophisticated model, in which the OFC encodes ‘task states’ by integrating stimulus-bound (external) and memory-based (internal) inputs. A central theme of this model is the ability of OFC to integrate disparate pieces of reward-related information in order to determine the ‘current state’, namely the current location on a cognitive map [27]. Recent experimental findings corroborated this concept by providing electrophysiological evidence that OFC encodes context-related information into value-based schemata, by showing that OFC ensembles encompass information about context, stimuli, behavioral responses as well as rewards associated with states [32]. Others have shown that blood oxygen level dependent (BOLD) functional magnetic resonance imaging (fMRI) signal, emitted by the OFC, correlates with reward value of choice in the form of a common currency that enables the discrimination between potential states based on their relative values [34, 35]. Valuation of states tend to occur automatically even if the cue is presented without the need for making decisions [36]. Further results posit that the OFC, rather than providing expected values *per se*, signals state values capturing a more elaborate frame about internal and external states including rewards, especially in the face of ambiguity [37]. The grave performance on tasks that mandate the disambiguation of states that are externally similar yet differ internally, when the OFC is impaired, points to the profound role this structure plays in creating new states (e.g. context frames) based on internally available information. Conversely, other lesion studies also implicated the significance of OFC in integrating contextual information into decisions, as human patients suffering from OFC impairment were shown to make irregular decisions, possibly because implications of the decision-making context were ignored, a behavioral finding that paralleled decreased BOLD signal in the related area [31, 38]. Contextual influence on decision-making is further captured by the framing effect, e.g. the contextual susceptibility of decision making, an effect that is also dependent on the intact functioning of the OFC [31].

OFC’s contribution to the other key element of the reward function, e.g. credit assignment, also has antecedents in literature. Credit assignment, one of the two domains determining action selection, is the association of behaviorally relevant stimulus with the action leading to preferable outcomes, by detecting state-action-state’ contingencies (as opposed to the policy domain that denotes choosing and implementing the most fruitful action from an available action set, see below) [39]. Credit assignment attributes value to a stimulus as a function of the precise history of actions and rewards with respect to the antecedent stimulus [40]. The OFC (in several reports: lOFC) has been identified as the structure that is responsible for credit assignment, as this subdivision was shown to conjointly encode recent history of state transitions and rewards, parallel to being able to alter the weight of an action that is indicative of the reward value in a given context [27, 33]. Single neuron recordings were also in line with credit assignment showing that the lOFC encodes the state transitions leading to the delivery of reward in a way that these representations are reactivated and maintained over different reward types [35]. Lesion studies implementing reward devaluation tasks offer similar insight, as macaques made fewer choices of the stimuli that signal the unsated reward, if lOFC was lesioned [41], a finding indicative of impaired credit assignment. That choices of the stimuli signaling unsated reward were less frequent upon lOFC lesions indicates the ability of the OFC to integrate cue- (e.g. the signal for reward), context- (e.g. internal context reflective of satiety) and action- (e.g. choosing the signal that indicates reward) related input. Conversely, Rushworth and colleagues have shown that OFC uses hippocampal/parahippocampal input to acquire and apply task-specific rules [35].

Implications that OFC conjointly signals information about reward identity, value, location, behavioral responses and other features [27, 42] was corroborated by works showing that OFC neurons encode all aspects of a task, they attribute rewards to preceding states and code state transitions [29, 37]. Prior experimental evidence has underlined the OFC neurons’ ability to exhibit outcome expectant activity based on afferent input, thereby signaling the value of outcomes in light of specific circumstances and cues [43]. This underscores OFC’s role in adapting to changing environments by enabling flexible behavior [43–47] facilitated by the formation of new associations between cues (states), state transitions and rewards via indirect links with other brain areas [33]. Using the Pavlovian over-expectation task, Takahashi and colleagues have revealed the critical contribution of OFC in influencing ongoing behavior and updating associative information by showing that reversible inactivation of the OFC during compound training omits the reduced response to individual cues [47]. Further support for the OFC, an essential part of the model-based reinforcement learning system, is reflected by the finding that lOFC lesioned animals, rather than crediting a specific cue or cue-action pair for the reward obtained, emit a signal characteristic of the recency-weighted average of the history of all reward received. Use of recency-weighted average to calculate the value of states is characteristic of model-free temporal difference learning [1, 3], allowing for the implication that, in the event, the model-based system is lesioned, the complementary model-free learning system will step in.

## Discussion

Albeit others have also formulated hypotheses that the OFC represents models for reinforcement learning, our proposition furthers this concept by linking a specific attribute of the Bellman equation descriptive of reinforcement learning to OFC function. A key new finding concerns the use of cue-context associations (deducted from the proactive brain concept) to explain OFC’s integrative function, with respect to cue- and context-related inputs (coming from the amygdala and hippocampus, respectively), reward expectations and credit assignment. Therefore we propose that the OFC computes the reward function attribute of the Bellman equation and thereby contributes to model-based reinforcement learning by assessing cue-context congruence along and maps cue/context/action-reward contingencies to context frames. By using the reward function, the OFC is able to signal predictions related to reward expectation.

To assess the specificity of our model we overviewed the function of other, significant interconnected structures implied in contributing to reinforcement learning, e.g. ACC, dorsolateral prefrontal cortex (dlPFC), pre-supplementary motor cortex (preSMC) and insular cortex [48, 49]. We found that their role may be well circumscribed and distinguished from the role attributed to the OFC by the proactive model of reinforcement learning. As proposed previously OFC’s role in reinforcement learning guided decision making concerns the ability to make detailed, flexible and adjustable predictions on context frames modelling the environment by assessing cue-context congruence and by means of credit assignment. With respect to ACC, its most commonly agreed upon feature is its engagement in decision making tasks that demand cognitive control. Two competing theories account for ACC’s distinct possible roles, with both acknowledging that ACC is involved in action selection based on the assessment of action-outcome relations [50–53]. Conversely it is involved in monitoring and integrating the outcome of actions [54]. The evaluative theory implicates that ACC monitors behavior to detect discrepancies between actual and predicted action outcomes in terms of errors and conflicts [50, 55]. Furthermore using the information about actual and predicted action outcomes, ACC may compute an index of unexpectedness, similar to the predicted error signal emitted by dopaminergic neurons, descriptive of the unexpectedness of actions [56]. The response selection theory, on the other hand, proposes that, rather than detecting or correcting errors, the ACC guides voluntary choices based on the history of actions and outcomes [51] by integrating reinforcement information over time to construct an extended choice-outcome history, with action values being updated using both errors and rewards [39].

In addition to governing the relationship between previous action history and next action choice, the ACC assumes a complementary role in exploratory generation of new action for the action set, used by reinforcement learning (this latter underlies the reinforcement potential of new situations) [39]. This is reflected by ACC’s role in foraging and other similar explorative behavior. Conversely ACC activation reflects estimates of the richness of alternatives in the environment by coding the difference between the values of unchosen and chosen options as well as the search value [57]. Lesion studies support ACC’s role in solving the exploration–exploitation dilemma reflected by impaired ability to make optimal choices in dynamically changing foraging tasks [51].

Summarizing, ACC is involved in one of the two domains of action selection, as it supplies information regarding the prospect of reward learnt from previous course of action (with the lOFC contributing to the other domain, credit assignment, reflective of behaviorally relevant stimuli [39]). An integrative theory of anterior cingulate function also postulated that the ACC is responsible for allocating control [58] by associating outcome values with different response options and choosing the appropriate action for the current environmental state [52, 59]. Using this information it directs the dlPFC and the preSMC to execute and implement the chosen action [52, 59, 60]. Analogous to the proposition that the mOFC computes the reward function of the Bellman equation, it may also be postulated that the ACC computes the policy function of the Bellman equation, respectively.

Regarding the involvement of ACC in reinforcement learning-based decision making it is also interesting to note that ACC (along with other structures like dlPFC and preSMC) is part of the intentional choice network (that is part of the larger executive network) [52]. Thus this higher level organization further supports ACC’s role in governing action selection in reinforcement learning. The insular cortex may be excluded from the line of model-free structures, given that it fails to meet axiomatic criteria prerequisite for model-free reward prediction error theory [48]. Nonetheless insular cortex’s contribution may be assessed in terms of model-based reinforcement learning, given its dense connections with model-based structures including amygdalal nuclei, OFC, ventral striatum, ACC and the dlPFC [61]. Its specific relationship with these structures is further augmented by the fact that connection is made by the outflow of a unique type of neurons called von Economo neurons [62]. In line with its functional connectivity, insula is responsible for detecting behaviorally salient stimuli and coordination of neural resources [60]. By means of its anatomical connections insula is able to integrate ascending interoceptive and viscerosensory inputs in a way that subjective feelings are transformed to salience signals influential of decision making [61]. Furthermore the anterior insula is implicated to be a key node, a ‘causal outflow hub’ of the salience network (that also includes the dorsal ACC) [63] that is able to coordinate two large scale networks, the default mode network and the executive network. The insula by emitting control signals via its abundant causal outflow connections is able to change the activation levels of the default mode network and the executive network, an effect formally shown by dynamic causal modeling of fMRI data [64]. Summarizing the insula has a central role in salience processing across several domains and is involved in mediating the switching between the activation of the default mode network and the executive network to ensure optimal response to salient stimuli [60] thus confers indirect, yet significant influence on model-based reinforcement learning.

It should be noted that albeit meticulous effort was made to associate each area with the most specific model-based reinforcement learning related attribute (e.g. mOFC: providing the model, lOFC: credit assignment, ACC: action selection, insular cortex: salience) there are reports that attribute other function to these structures (e.g. ACC and insular cortex coding reward prediction error signal [65, 66]).

### Computation of model-free reward prediction error hinges on input from the orbitofrontal cortex

**Fig. 1:**
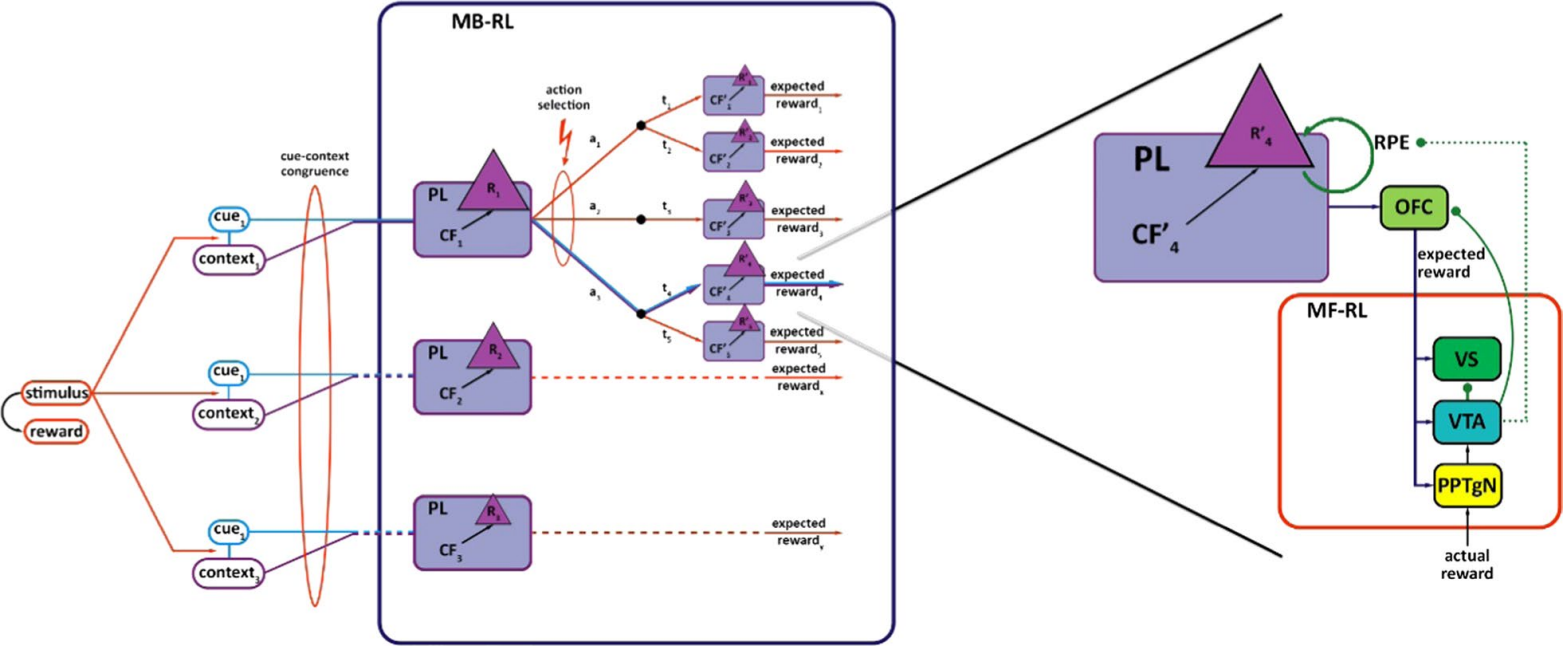
‘Proactive’ use of cue-context congruence for building reinforcement learning’s reward function. *Left panel* Salient stimulus, conceptualized as cue, and its context are processed by parallel but richly interconnected systems that center on the amygdala and hippocampus for cue-based and context-based learning, respectively. By means of Pavlovian learning, a set of relevant context frames are formed for each cue (hence, the uniform subscript of cues indicates the fact that a cue may be associated with distinct contexts, accordingly with distinct rewards). These context frames encompass permanent features of the context. Based on computational models of others and theoretical considerations, we presume that context frames also include reward-related information. According to the concept of proactive brain [23], when an unexpected stimulus is encountered, cue and context-based gist information is rapidly extracted that activates the most relevant context-frame that based on prior experience. Building on this, we propose that the reward function attribute of the world model is compiled by the OFC, which, by determining cue-context congruence, is able to identify the most relevant context frame. Using this context frame as a starting point (e.g. state), forward looking simulations may be performed to estimate expected reward and optimize policy (*dark blue line*). *Right panel* Upon activation of the most relevant context frame, predictions related to the expected reward will be made in the OFC. This information encompasses substantial environmental input and forwarded by glutaminergic neurons to the ventral striatum, VTA and PPTgN. The VTA will emit the reward prediction error signal, inherent of the model-free reinforcement learning system, by integrating actual reward and predicted reward information. In line with observations of others, we suggest that OFC derived expected reward information is incorporated into the reward prediction error signal (*dotted green line*). Furthermore, we propose that the scalar value of reward is updated by the reward prediction error signal contributing to the update of the world model. Abbreviations: action (a), context frame (CFx), model-based reinforcement learning (MB-RL), model-free reinforcement learning (MF-RL), Pavlovian learning (PL), reward (Rx), reward prediction error (RPE), transition (t), ventral striatum (VS), orbitofrontal cortex (OFC), ventral tegmental area (VTA), pedunculo-pontine-tegmental nucleus (PPTgN), *black dot* transitory state, *black arrow* glutaminergic modulation, *green arrow* dopaminergic modulation

Several testable hypotheses come from the bidirectional interactions between model-free and model-based learning. On one hand the OFC is known to project glutaminergic efferents to several structures involved in model-free reward prediction error signaling, including the PPTgN (that offers one of the strongest excitatory drives to the VTA [67, 68]), VTA [69] (that emits the model-free dopamine learning signal) and ventral striatum [16, 70] (that is responsible for computing value by compounding varying inputs (Fig. 1) [71, 72]). By reaching PPTgN, OFC may modulate the VTA’s most significant stimulating afferent, while OFC’s influence on dopaminergic neurons of VTA can extend to the alteration of both the spike and burst activity of dopaminergic neurons (e.g. presence of spike activity is prerequisite for burst firing). This anatomical connection is further supported by behavioral tests showing that the OFC’s reward expectation signal contributes to the detection of error in the reward prediction error signal, if contingencies are changing [43]. Relating experimental evidence, utilizing paradigms dependent on the update of error signals based on information about expected outcomes (e.g. the Pavlovian over-expectation task, Pavlovian-to-instrumental transfer, Pavlovian reinforcer devaluation and conditioned reinforcement), also pointed to the involvement of OFC [43]. Furthermore, expectancy-related changes in firing of dopamine neurons were shown to hinge on orbitofrontal input [37] as single unit recordings showed reciprocal signaling in OFC and VTA, which latter emits the prediction error during over-expectation tasks. This led to the conclusion that the OFC’s contribution to prediction errors is via its influence on dopamine neurons, as reward prediction single unit recordings in OFC were clearly related to the prediction error signal emitted by VTA [47]. Conversely, upon omitting the input from OFC, dopamine error signals failed to convey information relating to different states and resultant differences in reward [37].

This set of assumptions yield the hypothesis that the function of VTA dopaminergic neurons may be altered by cue-context manipulations leading to the change of glutaminergic input emanating from OFC, or by other interventions like transcranial magnetic stimulation.

### Updating the model by using model-free reinforcement learning signals

Another testable hypothesis concerns the use of model-free dopaminergic signal to update the model and action selection attributes of model based reinforcement learning. Linking our proactive model of reinforcement learning to the mathematical formalism of the Bellman equation gives a framework to jointly draw inferences concerning spatiotemporal environmental contingencies included in the reward function and action selection reflective of the reward structure contributing policy formation. As we have proposed, information about the scalar value of reward is encoded in context frames based on its spatiotemporal proximity with cues. This is done in a way that context frames may be mobilized based on cue-context congruence. Nonetheless it may be further inferred from our proactive model that feedback regarding the scalar value of reward, signaled as reward prediction error, may update the reward attribute of the cue-relevant context frame as follows. Neurobiological observations discussed previously show that, the main targets of VTA dopaminergic neurons are the ventral striatum (emitting the value signal that is characteristic of model-free learning), amygdala, hippocampus, OFC, ACC and insular cortex [48, 49, 70, 73, 74]. Considering the three factor rule, an extended form of the Hebbian rule, i.e. synaptic strength is increased if the simultaneous presynaptic and postsynaptic excitation coincides with dopamine release by means of long-term potentiation [75, 76], it may be postulated that in the event of dopamine release (the reward prediction error serving as a teaching signal) cue (amygdala), context (hippocampus) and cue-context congruence (OFC) relations are wired together, thus altering the reward structure (e.g. the environmental model). Therefore, the model-free reward prediction error output is necessary for updating the world model subserving the model-based system.

In addition, we have provided evidence that the ACC governs action selection and as such compiles the policy function. Conversely dopaminergic reward prediction error signals were also implicated to intervene with the process of action selection in the ACC. As it follows, the prediction error signal governs the decision, related to which of the several motor signals (available from the action set), should control the whole motor system [49], thus it determines action selection and as such updates the policy function.

Summarizing, this implication offers further indirect support for the interaction between model-free and model-based accounts by suggesting that model-free reward prediction error signal may contribute to updating the model used by model-based learning by altering the scalar value of rewards in the relevant context frames and it updates the policy underlying action selection to maximize outcomes.

### Clinical implications

The theoretical collision of the concept of proactive brain with that of reinforcement learning has substantial clinical relevance. A clinical exemplar, linking cue-context congruence to reinforcement learning concepts, comes from drug seeking behavior of addicts as it was shown that drug-paired contexts increase the readiness of dopaminergic neurons to burst fire upon encountering drug cues. This observation parallels dopamine’s tendency to prematurely respond to reward cues due to drug-induced alteration of the striatum. These effects could possibly be a net of altered OFC input to VTA and downstream structures that leads to the change of population activity and burst firing capacity of dopaminergic neurons [69]. Clinically, these observations may be related to the strong preference for drug-paired environments and cues in case of addiction, a phenomenon absent in non-addicts [77].

Furthermore proposing that reward-related information and action selection is governed by cue and context information (e.g. by the mobilization of the most relevant context frame based on cue-context congruence), we offer a framework for behavior modification. Given that reward information used by reinforcement learning depends on the statistical regularities of cue-context-reward co-occurrence, direct manipulation of cue-context-reward contingencies could overwrite former regularities to alter the reward function. Some currently used techniques of cognitive behavioral therapy (e.g. desensitization, chaining, triple or seven column technique) could be interpreted in terms of this framework. Furthermore, exploitation of technological advancements could be used to facilitate mental processes such as daydreaming or visualization [19] that contribute to the alteration of the model used by model-based learning. With the help of current technology, patients engage in activities in virtual settings, facing experiences that, according to our concept, would serve as input for shaping future behavior by formation of novel Pavlovian learning-based associations that alter existing spatio-temporal contiguities of cues, contexts and rewards, and may even extend to changes in state-state’ transitions.

## Conclusions

In summary, we put forward several testable hypotheses regarding how the brain handles model-based reinforcement learning. We postulated several structures of the model-based network to be involved in computing specific attributes of the Bellman equation, the mathematical formalism used to conceptualize machine learning based accounts of reinforcement learning. Furthermore we provided a plausible mechanism of how the model, used by model-based learning system, is created by organizing cue, context, reward information into context frames and capturing conjoint information of stimulus, action and reward. Furthermore based on the bidirectional interaction of model-free and model based structures we made two further proposition. One, given the reward value related input to the model-free structures (PPTgN and VTA), cue-context manipulations or transcranial magnetic stimulation may be applied to alter the model-free dopaminergic signal. Two, reward prediction error related dopamine signal may contribute to the update of both the model and the policy functions of model-based reinforcement learning. Furthermore our proactive framework for reinforcement learning has clinical implications as it builds on the use of cue-context associations to offer a representational architecture, upon which behavioral interventions may be conceptualized.

## Methods

The aim of the study was to provide a novel theoretical framework that formally links machine learning based concepts e.g. Bellman equation with the neurobiology of reinforcement learning and concepts of the proactive brain, by means of deductive reasoning. The merit of this concept is that it gives rise to several testable hypotheses and offers a representational architecture based on cue-context associations carrying clinical implications. The current work builds on our former work [4] and is based on conceptual and the experimental findings of others, cited throughout the text.

## Abbreviations

VTA: ventral tegmental area
PPTgN: pedunculo-pontine-tegmental nucleus
OFC: orbitofrontal cortex
mOFC: medial OFC
lOFC: lateral OFC
ACC: anterior cingulate cortex
dlPFC: dorsolateral prefrontal cortex
preSMC: pre-supplementary motor cortex
fMRI: functional magnetic resonance imaging
